# Concepts for regulation of axon integrity by enwrapping glia

**DOI:** 10.3389/fncel.2013.00256

**Published:** 2013-12-19

**Authors:** Bogdan Beirowski

**Affiliations:** Department of Genetics, Washington University School of MedicineSaint Louis, MO, USA

**Keywords:** axon, wallerian degeneration, schwann cell, oligodendrocyte, neurodegeneration, multiple sclerosis, amyotrophic lateral sclerosis, Charcot-Marie-Tooth disease

## Abstract

Long axons and their enwrapping glia (EG; Schwann cells (SCs) and oligodendrocytes (OLGs)) form a unique compound structure that serves as conduit for transport of electric and chemical information in the nervous system. The peculiar cytoarchitecture over an enormous length as well as its substantial energetic requirements make this conduit particularly susceptible to detrimental alterations. Degeneration of long axons independent of neuronal cell bodies is observed comparatively early in a range of neurodegenerative conditions as a consequence of abnormalities in SCs and OLGs . This leads to the most relevant disease symptoms and highlights the critical role that these glia have for axon integrity, but the underlying mechanisms remain elusive. The quest to understand why and how axons degenerate is now a crucial frontier in disease-oriented research. This challenge is most likely to lead to significant progress if the inextricable link between axons and their flanking glia in pathological situations is recognized. In this review I compile recent advances in our understanding of the molecular programs governing axon degeneration, and mechanisms of EG’s non-cell autonomous impact on axon-integrity. A particular focus is placed on emerging evidence suggesting that EG nurture long axons by virtue of their intimate association, release of trophic substances, and neurometabolic coupling. The correction of defects in these functions has the potential to stabilize axons in a variety of neuronal diseases in the peripheral nervous system and central nervous system (PNS and CNS).

## Introduction

Axons are the longest cellular projections of neurons relaying electrical and biochemical signals in nerves and white-matter tracts of the peripheral nervous system and central nervous system (PNS and CNS). As such, they are critical for neuronal wiring and transport of maintenance signals. The interdisciplinary study of how axons, together with the other neuronal and non-neuronal compartments in their entirety, contribute to connectivity and thus the great complexity of nervous system networks is a current effort at the forefront of neuroscience (see United States “BRAIN Initiative”, European “Human Brain Project”, and other similar programs worldwide).

**Figure 1:**
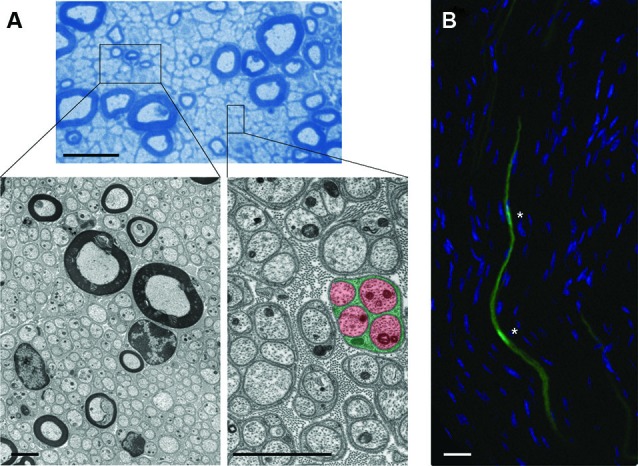
**Cytoarchitecture of EG and associated axons in mouse nerves. (A)** (Upper): Semithin microscopy of transverse section through mouse vagus nerve in which more than 90% of all fibers are unmyelinated and form multiple Remak bundles. Scale bar: 10 µm. Boxed areas from semithin preparation show electron micrographs (Lower) at different magnifications with characteristic cytoarchitecture of unmyelinating and myelinating SCs with their enwrapped axons. Note the tight association between glial processes (green) that interdigitate four unmyelinated small-caliber axons (red) in the highlighted Remak bundle from the right inset. Individual Remak bundles are surrounded by basement membranes and collagen fibers. Axonal and glial mitochondria are characterized by electron-dense appearance. Granular material within axons represents microtubules and neurofilaments. Scale bars: 2 µm. **(B)** Fluorescence confocal microscopy of longitudinal section through mouse tibial nerve (nuclear counterstain with 4’,6-Diamidino-2-Phenylindole (DAPI)) in which two adjacent SC bodies (green) are genetically labeled with green fluorescent protein (GFP). Asterisks depict nuclear regions of SCs with their elongated bodies. Scale bar: 5 µm.

Axons are enwrapped by glia with which they closely interact to form a unique symbiotic unit, a key contributor to the normal function of axonal connections. In vertebrate species, enwrapping glia (EG) are divided into Schwann cells (SCs) in the PNS and oligodendrocytes (OLGs) in the CNS, two distinct glial cell types with different morphologies and embryological origins (Nave, 2010a). EG are best known for insulating axons by encapsulating them with compact myelin to facilitate rapid saltatory impulse propagation (Jessen and Mirsky, 2005; Court et al., 2006; Salzer et al., 2008). Still, the majority of axons are not myelinated by EG in the PNS of higher vertebrates (Griffin and Thompson, 2008; Figure 1A), and compact myelination is completely absent in some vertebrates and most invertebrate organisms (Nave and Trapp, 2008; Rodrigues et al., 2011). Another well recognized but sometimes overlooked function of EG is the regulation of axonal structure, such as the control of axonal cytoskeleton composition and ion channel distribution (Salzer, 2003; Edgar and Garbern, 2004). In addition, there is an increasing appreciation that SCs and OLGs have various novel and previously unanticipated roles such as regulation of synaptic properties, control of the periaxonal ion microenvironment, immune-modulatory functions, and perhaps stem cell maintenance (Fields and Stevens-Graham, 2002; Griffin and Thompson, 2008; Martini et al., 2008; Zuo and Bishop, 2008; Yamazaki et al., 2011). Moreover, following injury, SCs orchestrate axonal regeneration in the PNS by promoting and directing axonal growth as well as restoring neuromuscular junctions (NMJs; Son et al., 1996; Chen et al., 2007; Sugiura and Lin, 2011). In reciprocity, distinct axonal signals regulate proliferation, survival, and differentiation of EG processes important for nerve and white matter tract development and repair (Barres and Barde, 2000; Jessen and Mirsky, 2005; Newbern and Birchmeier, 2010; Zuchero and Barres, 2013).

Pathological events in both SCs and OLGs, together with the common feature of compromised axon integrity, are implicated in a multitude of neurodegenerative diseases such as multiple sclerosis (MS; Bjartmar et al., 2003; Waxman, 2006), leukodystrophies (Garbern, 2007; Mar and Noetzel, 2010), Charcot-Marie Tooth neuropathies and hereditary spastic paraplegias (Suter and Scherer, 2003; Nave et al., 2007; Scherer and Wrabetz, 2008; Timmerman et al., 2013), and a series of acquired metabolic, inflammatory, and toxic neuropathies (Pardo et al., 2001; Meyer Zu Horste et al., 2007; Said, 2007). In many of these debilitating conditions, the culprit proteins and primary pathological events are predominantly or even exclusively found in EG. Nevertheless, aside from a narrow subset of cases where axonal damage is closely associated with demyelination and axo-toxic inflammation, it is poorly understood how defective EG contribute to axonal defects.

Importantly, early occurring axonal degeneration seems to be the main driver of clinical symptoms and morbidity in these conditions (Coleman, 2005; Nave et al., 2007; Taveggia et al., 2010). In MS, for instance, there is evidence for interrupted axonal continuity before the onset of demyelination, as well as following reparative remyelination (Trapp et al., 1998, 1999; Nikic et al., 2011; Manrique-Hoyos et al., 2012). Permanent neurological deficits result from axon damage because of the limited regenerative capacity of neurons (Compston and Coles, 2008; Trapp and Nave, 2008). Accordingly, delaying axonal attrition ameliorates disease in animal models for a number of neurodegenerative conditions associated with aberrant EG (Lo et al., 2002; Samsam et al., 2003; Bechtold et al., 2004, 2005; Kaneko et al., 2006; Chitnis et al., 2007; Meyer Zu Horste et al., 2011; Nikic et al., 2011).

There is now growing evidence that diminished support of EG for axons, independent of alterations in myelination, is an important event in the pathogenesis of above conditions (Edgar and Garbern, 2004; Nave and Trapp, 2008; Nave, 2010a,b; Morrison et al., 2013). Additionally, the ability of EG to support axons in nerves and white matter could have a general impact on the course of many other human neurological diseases. However, the nature of the axonal support function conferred by EG remains poorly defined. Clues for a better understanding may come from data on the role of injury responses in EG, glial release of trophic substances, and neurometabolic coupling between EG and axons. Equally obscure are the molecular mechanisms that govern the demise of long and vulnerable axons deprived of specific components from EG. Here, valuable information may be gained from advances in understanding experimental axon degeneration and its underpinning mechanisms.

In this review, I first elaborate on some of the major recent insights into axon degeneration models and its molecular regulation by cell- and non-cell autonomous pathways. I then attempt to illustrate the current state of knowledge on novel aspects of EG-axon communication which support the view that EG ensure long-term axonal integrity by blocking axonal auto-destruction programs. Combining these themes should encourage the development of a more integrated approach that addresses the cooperation between axons and their associated glia in neurodegeneration. In closing, I propose that dysfunctions of EG in conditions such as amyotrophic lateral sclerosis (ALS) and metabolic neuropathies should be viewed as central disease-modifying factors. Correcting these glial dysfunctions in combination with regimes to stabilize axons should open the way to novel therapeutic interventions for a broad range of axonopathies.

## Challenges for the maintenance of long axons

Axons are extremely long structures, with lengths and volumes often reaching 1000 times that of the parent neuronal cell body (Friede, 1963; Twiss and Fainzilber, 2009). This feature can be impressively demonstrated by long-range visualization of a small subset of PNS and CNS axons (Beirowski et al., 2004; Kerschensteiner et al., 2005). The extensive ramification or branching of axons in many neurons increases the total length and volume further (Matsuda et al., 2009). Correspondingly, axons have a very high energy demand, not least due to the requirement for maintenance and restoration of ion gradients, as well as the uptake and recycling of neurotransmitters. A resting corticospinal neuron, for example, consumes 4.7 billion ATP molecules per second (Zhu et al., 2012b). Given the relative sizes of different parts of the neuron, it is plausible to assume that this consumption occurs to a considerable extent in the axon. The enormous energy expenditure and the large volumes that are often spanned by axons between the cell body and the axon terminal at synapses confront neurons with a unique set of housekeeping challenges. These include, but are not limited to, the demand for uniform distribution of cytoskeletal constituents, energetic supply to distant axon regions, maintenance of calcium homeostasis, sequestering of aberrant axonal organelles and protein aggregates, endogenous protection mechanisms from mechanical and oxidative stress damage, and finally the need to transport various signals long distances from, and back to, the cell body.

**Figure 2:**
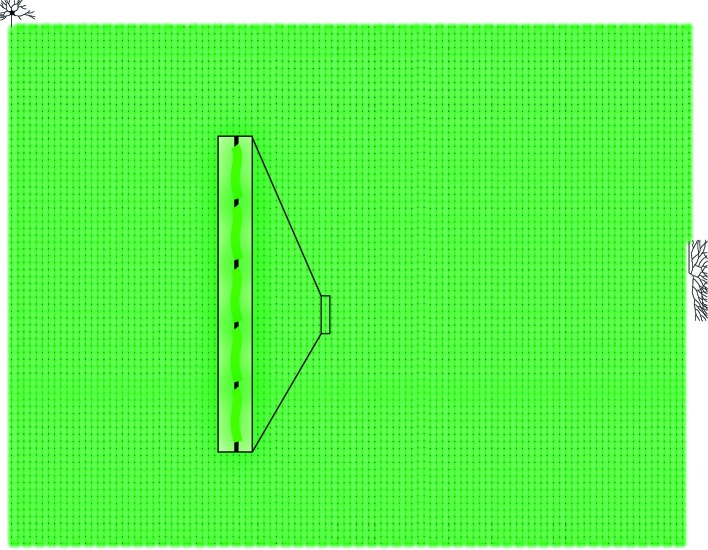
**Schematic illustration showing scaled model of human neuron/axon (black) in association with approximately 10,000 SCs (green) in proportion to size of neuronal soma and axon terminal.** Note that axons in humans or larger mammals often traverse distances of 1 m or longer and are accompanied by SCs almost over their full length. Inset shows higher magnification of boxed area in scaled model.

In light of these challenges, it is not surprising that axonal homeostasis is compromised in many diseases. In recent years, a large body of data has highlighted the importance of long-range bidirectional axonal trafficking defects of axonal proteins, mitochondria, vesicles and other cargo in their respective disease mechanisms (De Vos et al., 2008; Morfini et al., 2009). The identity of the upstream mechanisms leading to the observed transport decline, and the events that limit axonal health, however, often remain unknown. Because of their length and metabolic demand, axons are at continuous risk of damage, so it seems unlikely that cell-autonomous mechanisms are sufficient for their long-term maintenance. The unique cytoarchitecture with many thousands of EG closely flanking densely packed axons (Figures 1B, 2), suggests illustratively that EG may have an important role in exogenous axon support. On the other hand, the close proximity between axons and EG also suggests that even ephemeral pathological events in few EG may lead to focal axonal damage that could compromise the entire axon and therefore neuronal function.

## Cell-autonomous regulation of axon degeneration

Although axon degeneration and the associated changes in non-neuronal elements are such widespread events in neurodegeneration, we are just beginning to understand the underlying mechanisms involving cell- and non-cell autonomous pathways. Much of what we know about the cellular and molecular regulation of axon degeneration in pathological conditions originated in studies of experimental separation of PNS nerve fibers from their parent neuronal cell bodies, and in work on developmental axon elimination models (Coleman, 2005; Luo and O’Leary, 2005; Saxena and Caroni, 2007; Jessen and Mirsky, 2008; Wang et al., 2012a). In many species, nerve transection, a surrogate injury model for axon pathology in disease, results in rapid disintegration of axonal components (within circa 2 days), and a dynamic injury response in SCs and other cells distal to the transection site. “Wallerian degeneration” (WD) is often used as a more holistic term for this process to describe a collection of changes also in non-neuronal components (e.g., myelin collapse and ovoid formation, macrophage recruitment, breach of the blood-nerve barrier), and ensuing nerve remodeling in preparation for regeneration (Stoll et al., 1989; Vargas and Barres, 2007; Coleman and Freeman, 2010; Bosse, 2012; Rosenberg et al., 2012). Since axon degeneration patterns in chronic pathologies resemble this experimentally induced axon destruction pathway structurally and biochemically, it is referred to as “Wallerian-like degeneration” (WLD). WLD in disease has also mechanistically been compared to axonal pruning in development, on the grounds that both processes of axon degeneration share some molecular features and sometimes progress retrogradely towards the cell body (“dying back axonopathy”) (Spencer et al., 1976; Luo and O’Leary, 2005; Saxena and Caroni, 2007).

WD and WLD were initially believed to represent passive starvation or autolytic phenomena based on the assumption that compromised axon portions were deprived of their somatic nutritive sources (Vial, 1958; Schlaepfer, 1974; Lubinska, 1977). Intriguing in hindsight, it was proposed that EG can substitute for this nutritive function, given that in a number of invertebrates and some vertebrates severed axons can survive for months without their parent cell body (Bittner, 1991; Tanner et al., 1995). However, the discovery and initial study of the Wld^S^ mouse, in which WD of injured axons is dramatically delayed secondary to intrinsic alterations in axons because of the *Wld^S^* mutation (Lunn et al., 1989; Perry et al., 1990; Glass et al., 1993; Crawford et al., 1995; Coleman et al., 1998; Conforti et al., 2000; Mack et al., 2001), led to a paradigm shift and changed the way we think today about cell-autonomous mechanisms of axon degeneration. It is now clear that isolated rodent axon segments can exist and conduct electric signals for weeks without their cell bodies that bear the *Wld^S^* mutation or variants thereof (Tsao et al., 1994; Mack et al., 2001; Beirowski et al., 2009; Sasaki et al., 2009; Babetto et al., 2010). The Wld^S^ axon protection phenotype *in vitro* and *in vivo* is due to the ectopic expression of the aberrant Wld^S^ fusion protein which leads to the relocalization of nicotinamide mononucleotide adenylyltransferase 1 (Nmnat1) to axonal compartments (Beirowski et al., 2009; Babetto et al., 2010; Sasaki and Milbrandt, 2010; Cohen et al., 2012). Overexpression of the isoform Nmnat3 in mitochondria and Nmnat2 in axons also confers Wld^S^-like axon protection *in vivo* (Yahata et al., 2009; Milde et al., 2013a). Nmnat proteins (Nmnat1-3) are a group of critical enzymes in the biosynthesis of NAD^+^, which plays a vital role in energy metabolism, sirtuin biology, Ca^2+^ signaling, and other cellular functions (Berger et al., 2004; Lau et al., 2009; Ali et al., 2013). The exact working mechanism of Wld^S^ or Nmnat-mediated protection of axons is still essentially unknown, and a number of recent studies suggest a variety of occasionally contradictory models of action. The models involve modifications of axonal structure, organelles, and signaling under physiological conditions, the axonal site of Nmnat1 action, modulation of the mitochondrial permeability transition pore, a chaperone function of Nmnat1, bioenergetic alterations in axons containing excess Nmnat, and the involvement of other metabolic substrates besides NAD^+^ (Wang et al., 2005; Suzuki and Koike, 2007a,b; Press and Milbrandt, 2008; Wishart et al., 2008; Conforti et al., 2009; Sasaki et al., 2009; Yahata et al., 2009; Barrientos et al., 2011; Avery et al., 2012; Cohen et al., 2012; Fang et al., 2012; Shen et al., 2013). For a more comprehensive review on working mechanisms of Wld^S^ and axonal Nmnats, readers are referred to several recently published review papers on this subject (Coleman and Freeman, 2010; Yan et al., 2010; Fang and Bonini, 2012; Wang et al., 2012a).

Compellingly, Wld^S^ and its active component, axonal Nmnat1, not only delay WD after experimental nerve lesion, but also WLD and related pathologies in many mouse models of neurodegenerative disease (Wang et al., 2002; Ferri et al., 2003; Samsam et al., 2003; Gillingwater et al., 2004; Sajadi et al., 2004; Mi et al., 2005; Hasbani and O’malley, 2006; Kaneko et al., 2006; Howell et al., 2007; Beirowski et al., 2008, 2010; Meyer Zu Horste et al., 2011; Zhu et al., 2011, 2012a, 2013b). This suggests that axon degeneration after acute injury shares mechanisms with chronic axon loss occurring in neurodegeneration (i.e., WLD), and both possibly converge onto a common execution program for axonal dismantling. Two key components of this final pathway could be axonal transport failure and calcium influx that amongst other targets activates cysteine proteases such as calpains whose inhibition also confers protection of injured axons *in vitro* and *in vivo* (Ma, 2013; Ma et al., 2013). The aberrant Wld^S^ protein exerts its effects through a gain-of-function mechanism (Wld^S^ is not present in wild-type tissue) that can override the induction of pro-degenerative machinery in injured axons. Hence, axons should naturally be endowed with an endogenous, active and genetically controlled self-destruction program, similar to programmed cell death for the soma (Raff et al., 2002; Saxena and Caroni, 2007; Coleman and Freeman, 2010). This is plastically illustrated by spatiotemporal characterization studies demonstrating that WD occurs in a fast and explosive manner after a latency period, a process that can be blocked by Wld^S^ expression (Beirowski et al., 2004, 2005; Rosenberg et al., 2012).

In search for endogenous pathways regulating WD in wild-type axons, anterogradely transported Nmnat2 has been proposed as putative natural inhibitor of WD (Gilley and Coleman, 2010; Milde et al., 2013a,b). Endogenous Nmnat2 is short-lived and rapidly degraded following *in vitro* axotomy in cultured primary neurons, and depletion of Nmnat2 triggers rapid degeneration of uninjured neurites (Gilley and Coleman, 2010). Whether endogenous Nmnat2 has the same properties *in vivo*, and thus whether genetic deletion of Nmnat2 triggers spontaneous axon degeneration post-developmentally, however, remains an open question. The characterization of Nmnat2 knockout mice was reported recently, but these mice display severe peripheral axon outgrowth defects with perinatal lethality, precluding conclusions about the putative axon maintenance function of Nmnat2 (Hicks et al., 2012; Gilley et al., 2013).

While Nmnat2 acts as a negative regulator of WD, many endogenous positive regulators have also been recently revealed in genetic and pharmacological manipulations in mice, flies, and *in vitro* models through observation of loss-of-function effects (Miller et al., 2009; Barrientos et al., 2011; Gerdts et al., 2011; Wakatsuki et al., 2011; Bhattacharya et al., 2012; Osterloh et al., 2012; Xiong et al., 2012; Babetto et al., 2013; Gerdts et al., 2013). These discoveries point to the existence of an evolutionary conserved regulatory pathway that orchestrates the axon auto-destruction program after injury. The loss-of-function of some of these WD-promoting pathway components results in profound protection of experimentally lesioned axons in their respective mouse mutants much like that originally observed in Wld^S^ mice (Osterloh et al., 2012; Babetto et al., 2013; Gerdts et al., 2013). In contrast to modulation of axonal Nmnat levels, these targets may be more amenable to pharmacological manipulation. Of key importance, future studies must determine if these targets prove effective for axonal protection in mouse models for neurodegeneration, as this will elucidate whether they also have a regulatory role during WLD. Ongoing forward genetic screening studies involving high-throughput *in vitro* and invertebrate *in vivo* methods in several laboratories worldwide are expected to uncover further candidate constituents that control axon stability.

Additional active and cell-autonomous axon degeneration pathways, mostly implicated in developmental axon pruning models, are discussed in the following section focusing on the role of neurotrophic factors in axon stability. Although some molecules are shared with pathways governing WD (Low and Cheng, 2005; Luo and O’Leary, 2005; Hoopfer et al., 2006; Saxena and Caroni, 2007), separate mechanisms involving caspases appear to control them; interestingly, the canonical apoptotic program does not seem to be involved (Hyman and Yuan, 2012). While their role for WLD in disease is unclear, previous investigations demonstrate that apoptosis-like signatures can appear in chronic mammalian axon degeneration (Ahmed et al., 2002; Melli et al., 2006; Smith et al., 2011).

## Non-cell-autonomous mechanisms of axon dismantling during Wallerian degeneration (WD)

While the preceeding section discusses cell-autonomous mechanisms of axon degeneration disregarding the role of EG, there is emerging evidence that the rate of WD in the PNS can be substantially influenced by modulation of rapid pro-degenerative changes in SCs (Guertin et al., 2005; Parkinson et al., 2008; Woodhoo et al., 2009; Napoli et al., 2012; Yang et al., 2012). This implies that SCs might actively participate in the degenerative signaling events that execute axon and myelin dismantling in injured nerves, instead of simply sensing and cleaning up cellular debris as a passive bystander. Indeed, inhibition of pro-degenerative signaling in axon-flanking glia from *Drosophila* suppresses degeneration of genetically destabilized motor axons (Keller et al., 2011), similar to the protective effect of neuronal Wld^S^ expression in flies (Massaro et al., 2009).

It is well established that SCs sense axonal injury, form myelin ovoids, change their expression pattern towards an undifferentiated state (also referred to as “dedifferentiation”), and participate in the removal of axonal and myelin debris (Stoll and Muller, 1999; Hirata and Kawabuchi, 2002). Recent work has uncovered that a range of signaling events and transcription regulators in SCs, rapidly induced after nerve lesion, play an important role in this reactive SC response and the progression of WD (Harrisingh et al., 2004; Jessen and Mirsky, 2008; Parkinson et al., 2008; Jung et al., 2011; Arthur-Farraj et al., 2012; Fontana et al., 2012; Napoli et al., 2012; Yang et al., 2012). This opens the intriguing possibility that injured nerves and axons may be stabilized by non-neuronal manipulations. Remarkably, SCs can respond within a few minutes to axonal transection by activation of the ErbB2 receptor tyrosine kinase located at their ad-axonal membranes (Guertin et al., 2005). This is followed, within approximately 1 day, by induction of mitogen-activated protein kinase (MAPK) signaling including Ras/Raf and Erk1/2 kinases, p38 and c-Jun N-terminal kinase (JNK) (Harrisingh et al., 2004; Zrouri et al., 2004; Guertin et al., 2005; Agthong et al., 2006; Napoli et al., 2012; Yang et al., 2012), and further downstream actin polymerization at SC sites of non-compact myelin such as Schmidt Lanterman incisures (SLI; Jung et al., 2011). These events at SLI are known to regulate SC-mediated ovoid formation. Rapid induction of Notch signaling has also been demonstrated to accompany these early SC responses (Woodhoo et al., 2009). Whether cross-signaling between these pathways occurs in SCs is unknown. Strikingly, genetic or pharmacological blockade of these responses by MAPK, ErbB2 and actin polymerization inhibitors delays myelin breakdown and ovoid formation during WD *in vitro* and *in vivo* (Guertin et al., 2005; Woodhoo et al., 2009; Jung et al., 2011; Napoli et al., 2012; Yang et al., 2012). Conversely, induction of Notch or Raf/Erk signaling in SCs results in WD-like changes without the need to injure axons (Woodhoo et al., 2009; Napoli et al., 2012). While the authors suggest that experimental induction of these pro-degenerative SC responses does not trigger axonal injury within few days, it is not clear how these changes impact on axonal integrity over longer periods of time. Moreover, the fact that most of these ectopic signaling changes occur within approximately 1 day after nerve lesion suggests that they may precede the first structural signs of axonal breakdown during WD in the PNS (Lubinska, 1977; George and Griffin, 1994; Beirowski et al., 2005; Vargas and Barres, 2007). These signaling changes also appear to coincide with the latency or commitment phase of axon degeneration (Saxena and Caroni, 2007), suggesting that they might provide instructive signals for axonal breakdown. In support of this hypothesis, the substantially slower rate of axonal disintegration following experimental transection lesion in the mouse CNS (Beirowski et al., 2008, 2010; Babetto et al., 2013) versus the PNS (Beirowski et al., 2008, 2010) may be partially explained by the observations that similar pro-degenerative injury responses in OLGs in the CNS are weaker or absent (Hunt et al., 2004; Vargas and Barres, 2007). It appears that in the CNS microglia largely take over pro-degenerative response functions responsible for the eventual clearance of sick axons (Hosmane et al., 2012), but this occurs over a protracted time when compared to the removal of axons by SCs and invading macrophages in the PNS (Vargas and Barres, 2007).

It is known that the described intracellular signaling cascades in reactive SCs are able to control the expression and activation of various downstream transcription factors. Indeed, in addition to Notch regulation, the transcription factor c-Jun is rapidly upregulated after nerve injury in denervated SCs, an event that has been directly linked to the SC injury response and de-differentiation (Parkinson et al., 2008). Remarkably, the deletion of c-Jun selectively in SCs delays WD following nerve transection, largely by increased preservation of myelin sheaths. As a consequence, similar to Wld^S^ mice (Brown et al., 1992) (which fail to activate c-Jun), SC-specific c-Jun knockout mice display a dramatic inability to regenerate their nerves (Arthur-Farraj et al., 2012). Intriguingly, c-Jun activation exclusively in SCs in Wld^S^ mice is sufficient to destabilize distal transected axons in Wld^S^ mutants (Arthur-Farraj et al., 2012). In this way it is possible to abrogate the anti-regenerative signals present in preserved Wld^S^ nerve stumps.

While the implications of better understanding of cell-autonomous axon degeneration mechanisms are clear, the above data raise the question about the relevance of manipulation of SC injury responses in axonal disease. The injury-induced signaling cascades in SCs may have the capacity to modulate axonal stability in a non-cell-autonomous manner. Sick axons in disease are likely to elicit similar pro-degenerative SC injury responses that could be detrimental to axons by reciprocal amplification of damage over time. Such axons may have not yet entered a commitment phase of degeneration, and their damage may thus be potentially revertible. Furthermore, reactive SCs prominently trigger inflammatory signals that attract macrophages with other immune cells (Napoli et al., 2012). In an environment of densely packed axons, this might compromise uninjured neighbor axons. Thus, modulation of the SC injury responses may serve as a therapeutic approach to limit the expansion of axon damage in disease. Of note, increased levels of c-Jun in nerve specimens from patients with peripheral axonal neuropathies have been recently reported (Hutton et al., 2011), and high levels of ErbB2 and MAPK signaling activation with inflammatory responses have been documented in peripheral neuropathy situations (Tapinos et al., 2006; Kohl et al., 2010). It is not known if these glial injury responses are the causes or effects of axonal damage in these conditions. Further studies are required to determine the temporal relationship between axonal damage and occurrence of the first signs of glial injury response, and it is necessary to test the therapeutic potential of blocking these responses. Even if pro-degenerative responses in SCs follow axon damage in these conditions, the therapeutic restriction of axon-glia interactions resulting in reduction of inflammatory events may limit axonal damage.

Taken together, recent advances demonstrate that axon degeneration after distinct insults is an active and tightly orchestrated self-destructive process. The discovery of neuronal gain and loss-of-function mutations that inhibit axon degeneration opens for the first time the possibility to stabilize diseased axons, so far a therapeutically unexploited area in neurodegenerative conditions. Additionally, there is now evidence showing that dismantling of injured nerves is also dependent on non-cell-autonomous mechanisms in form of a pro-degenerative SC injury response. The mechanistic relationship between endogenous axonal auto-destruction and the pathways regulating these glial responses is unknown, a problem that begins with the lack of understanding how EG sense axonal injury. Moreover, it is unclear if glial reactive responses could drive axonal destabilization in chronic disease conditions. In sum, these considerations blur the line between neuronal and non-neuronal pathways regulating integrity of the axon-glia unit.

## Depletion of enwrapping glia (EG) results in compromised axon integrity

The hallmark role of EG in axonopathies and the behavior of SCs in response to axonal injury invites the question of which functions EG generally have for axon stability under physiological conditions. First, illustrative in a simplified model, it is clear from innumerable culture studies that maintenance and functionality of PNS and CNS neurons and their processes is markedly extended if they are cultured together with EG or other glial cells, or after administration of glia-conditioned medium (Selak et al., 1985; Takeshima et al., 1994; Meyer-Franke et al., 1995; Sortwell et al., 2000; Wender et al., 2000; Gardner et al., 2012). Second, a number of rodent *in vivo* models have been developed providing a valuable resource to investigate the consequences of genetic or cytocidal ablation of EG for axon integrity. Depleting SCs during development by ErbB2 or ErbB3 deletion does not grossly impair initial muscle innervation, but the absence of SCs results in dramatic degeneration of mouse ventral root axons and terminal axon portions at NMJs, prompting the idea that EG could promote the trophic support of axons (Riethmacher et al., 1997; Woldeyesus et al., 1999; Lin et al., 2000). In fact, axon terminal degeneration in mice also can be triggered by exogenous neuregulin manipulation (neuregulin is a potent SC migration stimulator) that leads to departure of perisynaptic SCs from NMJs (Trachtenberg and Thompson, 1997). Similarly, ablation of such SCs at NMJs by complement-mediated cell lysis in tadpoles, results in retraction and degeneration of axon terminals (Reddy et al., 2003). Regarding sensory fibers, selective apoptotic reduction of non-myelinating SCs in mice elicited through disruption of ErbB receptor signaling leads to degeneration of small diameter nociceptive axons (Chen et al., 2003). Intriguingly, similarly progressive degeneration of exclusively small sensory axons occurs after disruption of fibroblast growth factor 1 and 2 (FGF-1, 2) signaling in SCs (Furusho et al., 2009).

Recently, several groups tested the consequences of targeted ablation of OLGs in the mouse CNS. Diphtheria toxin (DT)—mediated death of exclusively OLGs results in CNS axon degeneration in form of fiber dystrophy (Ghosh et al., 2011a; Pohl et al., 2011; Oluich et al., 2012). Importantly, OLG death and concomitant axon damage can occur in the absence of overt demyelination and independent of immune activity excluding the possibility that axon damage is triggered mostly by inflammatory processes (Pohl et al., 2011; Oluich et al., 2012). This corroborates the notion that CNS axons require the physical presence of OLGs for preservation of their integrity.

Collectively, these sets of *in vitro* and *in vivo* observations indicate that axons cannot exist in isolation without their EG. This suggests that substances released from EG are essential for axonal function and integrity.

## The role of myelination for long-term axonal maintenance

An intriguing and recurrent debate regards whether the myelin coat of axons may be beneficial for maintenance of their integrity. It is founded on a series of classical studies involving experimental manipulation of myelin formation, extensive research on myelin-specific inherited and autoimmune diseases, and more recently on the observations that axonal transport failure and WLD occur in mutant mice that lack specific myelin proteins (a comprehensive review of the literature is provided in Nave, 2010a,b).

EG derived myelin is a multilamellar, tightly compacted membrane that consists largely of lipids and proteins (Chrast et al., 2011). Proteolipid protein (PLP) is the major myelin protein in the CNS while protein zero (P0) is the major constituent in PNS myelin (Yin et al., 2006). Myelin is sometimes viewed as a protective sheet that shields axons from exposure to harmful extracellular milieu, a property that is compromised in demyelinating diseases. Accordingly, the axonal degeneration observed in some PNS hypomyelinating mutants was believed to be due to absence of myelin (Rosenfeld and Freidrich, 1983; Barron et al., 1987). It is, therefore, often presupposed in the current literature that myelin itself is axoprotective (e.g., Tawk et al., 2011). This could be especially true for large diameter axons, as during transmission of action potentials the compact myelin sheath clearly reduces axonal energy consumption (Rosenbluth, 2009). Thus, bioenergetic requirements may be limiting for long-term preservation of demyelinated large axons in disease. However, it was also speculated that myelin may provide axonal support through embedded trophic molecules (Bjartmar et al., 2003; Yin et al., 2006; Wilkins et al., 2010). Yet, experimental blockade of compact myelin formation in the PNS and CNS did not result in WLD as seen in demyelinating pathologies, despite the observation of reduced axonal diameter (Aguayo et al., 1979; De Waegh et al., 1992; Colello et al., 1994).

There appears to be an increasing consensus now that the postulated axon support function of SCs and OLGs is independent from compact myelination although the removal of myelin can have detrimental consequences for axons (Edgar and Garbern, 2004; Nave and Trapp, 2008; Nave, 2010a,b; Morrison et al., 2013). In other words, although a myelinotrophic effect for overall axon maturation is unquestionable, the absence of myelin as such does not necessarily impair axonal survival. For example, Shiverer mice (deficient for myelin basic protein (MBP)) do not show loss of any axon population despite virtual absence of myelin (Rosenbluth, 1980a,b; Griffiths et al., 1998), and display increased instead of decreased axonal transport components (Kirkpatrick et al., 2001). Similar findings with no axon demise have been recently obtained in myelin-depleted shaker rats even in aged animals (Smith et al., 2013). On the contrary, deletion of only PLP from CNS compact myelin results in defective axonal transport (Edgar et al., 2004), and robust axon degeneration in form of axonal swelling and continuity interruption (Griffiths et al., 1998; Garbern et al., 2002; Yin et al., 2006). Strikingly, the PLP loss does not have much consequence for the formation of compact myelin (Klugmann et al., 1997; Rosenbluth et al., 2006) indicating that this genetic intervention decoupled the role of OLG for myelin formation from its role for axonal maintenance. The appearing axonal swelling or dystrophy in absence of PLP is a hallmark of WD and WLD in the CNS (Coleman, 2005; Beirowski et al., 2010) which can be blocked by Wld^S^ axon protection in other chronic axon degeneration models (Mi et al., 2005).

Moreover, 2′,3′-Cyclic nucleotide 3′- phosphodiesterase (CNP), a myelin protein with the ability to enzymatically convert 2′,3′-cyclic adenosine monophosphate (cAMP) to 2′-adenosine monophosphate (AMP) and thereby promote production of adenosine, is equally essential for normal long-term axonal integrity, but dispensable for myelination, as mice with a targeted disruption of the *CNP* gene develop late-onset CNS axonal degeneration despite only marginal myelin abnormalities (Lappe-Siefke et al., 2003). The additional deletion of PLP in these mutants substantially aggravates the axonal demise, but not myelin defects (Edgar et al., 2009). Finally, grossly normal myelin can be formed in the absence of enzymes important for synthesis of specific myelin lipids, but surprisingly this results in axon degeneration (Sheikh et al., 1999; Zoller et al., 2008). The mechanisms of how absence of these myelin proteins and lipids causes axonal degeneration have not been identified, but biochemical screening studies suggest that some of these proteins may facilitate the transport of trophic substances from OLGs that could be important for axonal integrity (Werner et al., 2007). Alternatively, in the case of CNP deletion for example, an increased content of toxic 2′,3′-cAMP or diminished production of neuroprotective adenosine in OLGs may be at least in part responsible for axonal destabilization (Verrier et al., 2013).

As a whole, in recent years it became increasingly clear that the postulated axon-support function of EG is independent of myelination *per se*. Rather, it appears that specific myelin components produced in EG are required for long-term axonal preservation. The underpinning mechanisms remain to be identified, and it is unclear if these constituents play also a role for the survival of peripheral axons. On the other hand, inflammatory removal of myelin in demyelinating conditions such as MS may destabilize axons by leaving them exposed to injury by deleterious immune components. Independently, demyelination can also contribute to axonal degeneration through aberrant axonal domain organization and impairment of axonal transport (Salzer, 2003). Thus, remyelination is a favored goal in these conditions (Franklin and Ffrench-Constant, 2008).

## Correct axon-glia contact is essential for axonal support by enwrapping glia (EG)

A logical prerequisite for trophic support of axons by EG is correct physical alignment between these two cell types, especially at sites of specialized contact between the axolemma and the ad-axonal membrane of EG. These axo-glial junctions are locations of highest probability for delivery of trophic substances. In addition, correct arrangement of putative nutritive channels of non-compact myelin between axons and myelinating EG should be crucial, a feature inextricably linked to myelination. These may include structures such as the inner mesaxon, SLIs, and paranodal cytoplasmic loops (Duncan et al., 1996; Lopez-Verrilli and Court, 2012). Consequently, severance of such transport systems and impaired membrane contact by defects in molecules mediating cellular adhesion could result in axon instability because of the failure to transfer nutritive substances from the EG into the axon. Such disruption may occur in chronic axonopathies as a consequence of extensive demyelination, or perhaps alongside only subtle changes in the myelin architecture secondary to mutations in structural proteins.

A number of studies have documented evidence for this model. Physical interaction between EG and axons and regulation of axo-glial junction formation in myelinated fibers are mediated by various adhesion molecules such as L1, myelin-associated glycoprotein (MAG), the Necl proteins, NrCam, neurofascin 155 and 186, Neural Cell Adhesion Molecule (N-CAM), N-cadherin, Caspr (NCP1), contactin and their associated scaffolding proteins (Poliak and Peles, 2003; Salzer et al., 2008). Deletion of several of these proteins *in vitro* and *in vivo* results in aberrant axo-glial contact, reduced adhesion between axolemmal and ad-axonal membrane domains, and eventually in axon degeneration (Scotland et al., 1998; Yin et al., 1998; Haney et al., 1999; Pan et al., 2005; Garcia-Fresco et al., 2006; Buttermore et al., 2011; Golan et al., 2013). For instance, L1 deficient mice develop progressive loss of unmyelinated sensory axons owing to reduced axonal attachment to SC processes (Haney et al., 1999). Notably, loss of axonal L1 does not lead to failure to myelinate or maintain large fibers, suggesting that axonal L1 is specifically required to mediate appropriate contact between non-myelinating SCs and their axons. In contrast, MAG appears to be necessary for maintenance of contact between myelinating SCs and large diameter axons in adult animals, as MAG knockout mice develop late-onset axonal detachment from EG and consecutive axon degeneration, while producing comparatively normal myelin (Yin et al., 1998; Pan et al., 2005). Intriguingly, expression of MAG also stabilizes injured axons during WD and following other axonal insults (Nguyen et al., 2009). Thus, the early disturbance of MAG expression together with other adhesion molecules in chronic axonopathies may contribute to axonal destabilization (Kinter et al., 2012). Moreover, Caspr mutant mice develop aberrant paranodal junctions, axonal transport defects of mitochondria in sciatic nerves, and dystrophic degeneration of long Purkinje cell axons (Einheber et al., 2006; Garcia-Fresco et al., 2006). These findings support the idea that proper structure of nutritive channels in paranodes is essential for support of axons.

In short, these reports underscore the importance of tight apposition and correct configuration of critical membrane domains of EG and axons to ensure delivery of trophic substances and thus the long-term maintenance of the axon.

## The role of neurotrophic factors in the support of axons by enwrapping glia (EG)

Correct neurotrophic factor signaling is absolutely necessary for the function of the nervous system, including normal development, differentiation, and survival, as well as synaptic plasticity and transmitter release. Because EG produce and release various neurotrophic factors, and these molecules have been implicated in axonal stability in the last years (see below), it is important to evaluate their roles in the context of axon-glia interaction. Neurotrophic factors can be divided into the categories neurotrophins, the glial cell-line derived neurotrophic factor (GDNF) family, neurokines (e.g., ciliary neurotrophic factor (CNTF)), and other pluripotent growth factors (e.g., Insulin-like growth factor 1 (IGF-1)). Neurotrophic functions relevant for neuronal health are best studied for the neurotrophin family members nerve growth factor (NGF), NT3, NT4/5, and BDNF, which are target-derived growth factors that signal through the TrkA, B, and C members of the receptor tyrosin kinase family, and through the common low-affinity P75^NTR^ receptor (Chao, 2003). Further, secreted neurotrophins and other growth factors and mitogens orchestrate various stages of EG development and myelination by acting upon their receptors expressed in EG itself or by eliciting secondary axonal signals, a function studied mostly in *in vitro* models (Rosenberg et al., 2006).

In neurons, following interaction with their receptors (on the axonal surface), many neurotrophin signals are propagated retrogradely along the axon by endosomal trafficking of macromolecular complexes towards the cell body in order to exert their biological effects in neuronal compartments (Harrington and Ginty, 2013). This is not only important for neuronal development and maintenance under physiological conditions, but also for appropriate response to neuronal injury to initiate regeneration. Studies of cultured primary neurons and a multitude of knockout mouse models for neurotrophic factors demonstrated the importance of the downstream pathways for the suppression of developmental apoptosis, for example by transcriptional upregulation of pro-survival genes in neurons. Because of the overall degeneration of all neuronal portions after neurotrophic factor deprivation in this setting, a maintenance role for these factors on the axonal compartment is generally difficult to deduce. However, a direct role for selective axon maintenance is supported by circumstantial evidence. For example, although accompanied by premature lethality in the majority of mutants, surviving NT3 knockout mice initially form normal appearing motor innervation, which is rapidly followed by catastrophic breakdown of intramuscular nerve branches and axon terminals (Woolley et al., 2005). Remarkably, the nerve degeneration visualized by neurofilament labeling closely resembles paradigms of experimental axon degeneration after nerve cut. This suggests that NT3 is not necessary for development of a subset of motor neurons and their axons, but later required for axonal preservation. Other *in vivo* support for a local action of individual neurotrophins for axon stability comes from research on peripheral neuropathies that are secondary to mutations in neurotrophin-retrograde-transport machinery such as the small GTPase RAB7 implicated in axonal Charcot-Marie-Tooth type 2B disease (Deinhardt et al., 2006; Zhang et al., 2013).

In the last years significant independent evidence has accumulated supporting local neurotrophic factor function in axons, primarily that of neurotrophins and CNTF, which control axonal integrity. By developing a compartmentalized chamber system in which the somatic portion of the neuron is divided from the axon, the Campenot laboratory demonstrated that local withdrawal of NGF from the axonal compartment results in degeneration of the sympathetic axons, with no immediate effect on survival of the neuronal cell body (Campenot, 1994). Recently, an emerging body of literature has more closely implicated neurotrophin signaling in the control of axonal degeneration, and elucidated some of the downstream signaling events following withdrawal. NGF deprivation (or withdrawal of other neurotrophins) in axons from primary neurons in compartmentalized chambers or microfluidic culture systems simulates a degeneration process often referred to as axonal pruning. This is considered a model for developmental axon degeneration meant to remove superfluous neuronal projections, but also for WLD in disease (Raff et al., 2002; Luo and O’Leary, 2005; Saxena and Caroni, 2007). Using sensory or sympathetic axons, a number of reports demonstrated the involvement of selective apoptotic pathway components (mainly caspase 3, 6 and 9 downstream of the death receptor DR6) in the local regulation of this mode of axon degeneration (Nikolaev et al., 2009; Schoenmann et al., 2010; Vohra et al., 2010; Simon et al., 2012; Uribe et al., 2012; Cusack et al., 2013; Unsain et al., 2013). The mechanisms that ultimately lead to activation of this apoptosis-like system are largely unknown. It has been recently shown, however, that degeneration of axons induced by neurotrophic factor deprivation is regulated by signaling pathways such as Dual leucine zipper kinase (DLK) and glycogen synthase kinase-3β (GSK3β) signaling, specific transcriptional regulators, local protein synthesis of axonal survival factors (Impa1, LaminB2, B-cell lymphoma w (Bcl-w)), and the ubiquitin proteasome system (Watts et al., 2003; Macinnis and Campenot, 2005; Andreassi et al., 2010; Ghosh et al., 2011b; Wakatsuki et al., 2011; Chen et al., 2012b; Yoon et al., 2012; Cosker et al., 2013). Importantly, some of the discovered pathways in culture model systems appear to be relevant for chronic axon degeneration models *in vivo* (Courchesne et al., 2011; Smith et al., 2011).

Conversely, neurotrophins can also be axon-destructive suggesting a disparate nature, similar to what is reported about opposing neurotrophin effects on cell survival or glial development (Chan et al., 2004). This initially has been shown in an *in vivo* model to study development of sympathetic and olfactory sensory axons, in which activity-dependent release of BDNF by axons caused degeneration of competing neighbor axons via activation of p75^NTR^ (Cao et al., 2007; Singh et al., 2008). Before that, a function of p75^NTR^ in axonal degeneration has also been proposed based on studies utilizing p75^NTR^ overexpression in cultured neurons derived from embryonic stem cells (Plachta et al., 2007). Moreover, BDNF binding to p75^NTR^ in adult septal cholinergic axons can induce local degeneration mediated by axonal caspase-6 activation, and BDNF antibodies inhibit this effect (Park et al., 2010).

It is widely appreciated that SCs are important providers of growth factors including neurotrophins, and that the release is dynamically and differentially regulated in distinct SC populations especially following experimental nerve transection (Sendtner et al., 1992b; Funakoshi et al., 1993; Friedman et al., 1996; Frostick et al., 1998; Brushart et al., 2013). First, neurotrophic factor secretion from SCs considerably supports axonal growth and provides important guidance cues for regenerating fibers to reach appropriate peripheral targets (Chen et al., 2007). Second, neurotrophins released from SCs have a vital role in the regulation of pathways leading to myelination (Rosenberg et al., 2006; Xiao et al., 2009). One of the most notable pathways was discovered in *in vitro* studies investigating the function of SC-derived NGF and BDNF, which were shown to stimulate myelination of DRG axons via p75^NTR^ or Trk receptors, while NT3 regulates myelination negatively via TrkC on SCs (Chan et al., 2001, 2004; Cosgaya et al., 2002). Together, these functions form the basis for therapeutic interventions by SC transplantation in the injured rodent CNS (Xu et al., 1997; Hu et al., 2005).

Much less interpretable data is available on the role of SC-derived neurotrophic factors for long-term axonal maintenance under basal conditions and during chronic neurodegeneration. SCs clearly are a source of growth factors that largely can affect the survival of neurons. For example, constitutive neurotrophin secretion occurs in cultured SCs, and this extends neuronal viability, which is also enhanced if neurons are treated with conditioned SC medium containing these neurotrophins (Taylor and Bampton, 2004; Thippeswamy et al., 2005). Moreover, following spinal cord repair, expression of NT3 and other neurotrophins in SCs sustains survival of newly regenerated axons, and reduction of NT3 results in axonal die-back (Hou et al., 2012). It has been speculated that axonal destabilization in peripheral neurodegeneration may be due to reduced delivery of neurotrophic factors from SCs into axons (Friedman et al., 1996; Haney et al., 1999; Gatzinsky et al., 2003; Nobbio et al., 2009). Indeed, a decline in SC-derived IGF-1 and other growth factors accompanying axon degeneration was recently reported in a mutant superoxide dismutase 1 (SOD1) mouse model for ALS (Lobsiger et al., 2009). Furthermore, reduced expression of CNTF in SCs is observed in the most common form of hereditary peripheral neuropathy (Charcot-Marie-Tooth Disease type 1A (CMT1A)), which is characterized by axonal atrophy (Friedman et al., 1996; Nobbio et al., 2009). CNTF, in contrast to other factors not necessary for neuron development, is predominantly released by SCs (the release mechanism is essentially unknown), and whole-body genetic ablation of CNTF in mice results in early degeneration of facial motor axons (Masu et al., 1993). The motor axon degeneration is preceded by axonal atrophy and abnormalities at juxtaparanodal junctions between SCs and axons (Gatzinsky et al., 2003). In line with an axonal maintenance role, CNTF administration blocks motor axon degeneration in pmn mice (Sendtner et al., 1992a), and in the mutant SOD1 mouse model for ALS (Pun et al., 2006), two paradigms with early occurring distal axon damage. Mechanistically, it was recently demonstrated that CNTF acts locally in sick motor axons to control microtubule stability via the Janus kinase-Signal Transducer and Activator of Transcription 3 (JAK-STAT3) pathway and stathmins (Selvaraj et al., 2012). Finally, erythropoietin (EPO) released from SCs appears important for axon protection (Keswani et al., 2004), and it is possible that EPO both functions by reducing SC pro-degenerative responses in injured nerves, and by increasing axon stability (EPO receptor is present on axons as well as on SCs) (Li et al., 2005; Campana, 2007). Remarkably, administration of EPO and induction of signaling leading to its enforced expression ameliorates axon pathology induced by acrylamide *in vivo*, while inhibition of SC-derived EPO makes axons more vulnerable to neurotoxic paradigms (Keswani et al., 2004, 2011). In view of these results, axo-protective EPO therapy and application of other neurotrophic factors has been suggested for treatment of hereditary peripheral neuropathies (Nave et al., 2007).

Similar to SCs, OLGs excrete growth factors such as NGF, NT3, BDNF, GDNF, and IGF-1, with a proposed trophic support role for various CNS neurons, and also for their axonal compartments under distinct experimental circumstances (Wilkins et al., 2001, 2003; Du and Dreyfus, 2002; Dai et al., 2003; Wilkins and Compston, 2005; Smith et al., 2013). Based on exclusion experiments with specific inhibitors and blocking antibodies, Dai et al. (2003) proposed that other yet-to-be identified trophic substances sustain neuronal health (Dai et al., 2003). One of these may be FGF-9, whose expression has been reported in adult OLGs *in vivo* (Nakamura et al., 1999). The above data do not fully resolve the question of whether the trophic effects nourish the axonal or the cell body compartment or both, but they are certainly consistent with a possible function in axonal support. Future experimentation is needed for characterizing their role in more detail, for example by employing precise spatiotemporal analysis of axon degeneration *in vivo* following controlled withdrawal or block of these factors from OLGs.

Taken together, work from the last few years unveiled a central role of neurotrophic factors for axonal maintenance, predominantly in *in vitro* models. Axons seem to engage an apoptosis-like cascade following local deprivation or sometimes elevation of these factors. Although considerable evidence underscores an important function for release of neurotrophic factors from SCs and OLGs for axonal growth and myelination, their precise role as EG-derived axonal maintenance factors remains to be determined. Future studies, employing inducible EG-specific deletion for neurotrophins and other growth factors *in vivo*, and compartmentalized culture investigations *in vitro*, may elucidate these open points.

## Support of axons by enwrapping glia (EG) via vesicular transport of trophic substances

Long axons may not only depend on delivery of neurotrophic factors, but also on other macromolecules from adjacent glia. Since there is no evidence for intercellular cytoplasmic junctions between EG and axons (Nualart-Marti et al., 2013), transport of extracellular microvesicles that contain trophic components is a prime candidate (Simons and Raposo, 2009). Exchange of such vesicular vectors has emerged as key factor for intercellular communication in the last decade, being involved in the regulation of a diverse range of biological processes. One of the first clues for vesicular transport from EG into axons came from studies indicating transfer of labeled proteins, lipids, and RNA from axon-flanking glia into anucleated giant axons from squid, crayfish, and goldfish (Jakoubek and Edstrom, 1965; Lasek and Tytell, 1981; Gould et al., 1983; Tytell et al., 1986). As mentioned above, such injured axons can survive for months, a feat that may partially be explained by postulates that squid glial cells replenish the axonal protein pool by transferring as much as 40% of their newly synthesized proteins to the giant axon (Lasek and Tytell, 1981). One particularly interesting group of transported proteins are heat shock proteins (HSPs), which a study in crayfish suggested may protect axons from stress and injury (Sheller et al., 1998). Meanwhile, migration of fluorescently labeled vesicles from glia into the squid giant axon were observed (Buchheit and Tytell, 1992), suggesting a special mode of exchange exists between EG and the axon. Subsequently, similar observations and interpretations were made in the PNS and CNS of vertebrates including rodents (Campbell and Peterson, 1993; Edbladh et al., 1994; Duncan et al., 1996).

Research from the past few years has given significant insight into the nature of a particular class of EG-secreted vesicles, and into their neuronal and axonal internalization (Kramer-Albers et al., 2007; Hsu et al., 2010; Bakhti et al., 2011; Fruhbeis et al., 2012, 2013; Lopez-Verrilli and Court, 2012). We now know that at least OLGs follow an unconventional secretory pathway by releasing lipid-bilayer enclosed exosomes, 50–100 nm large vesicles derived from late endosomes and multivesicular bodies (MVB), that contain a wide array of putative trophic components (Kramer-Albers et al., 2007; Hsu et al., 2010; Bakhti et al., 2011). The secretion occurs in a calcium-dependent manner and is facilitated by neuronal activity via glial ionotropic glutamate receptors (Fruhbeis et al., 2013). Exosome internalization and functional incorporation of the content by neurons recently has been demonstrated *in vivo* (Fruhbeis et al., 2013). Intriguingly, many of the proteins identified by mass spectrometry in these exosomes are enzymes involved in carbohydrate and lipid metabolism and components previously involved in neuroprotection such as chaperones, HSPs, enzymes that help reducing oxidative stress, and the sirtuin Sir-two-homolog 2 (SIRT2; Kramer-Albers et al., 2007). This cytoplasmic NAD^+^ dependent sirtuin has been recently implicated in cell-autonomous axonal stability mechanisms, toxin-induced axon degeneration, and the pathways mediating the Wld^S^ phenotype (Suzuki and Koike, 2007a,b; Wishart et al., 2012). Moreover, exosomes contain cytoskeletal elements, ribosomal constituents, and RNA possibly together with microRNA species. Arguing for a neuronal maintenance role, it was further shown that the administration of exosomes from OLGs to cultured neurons supports their metabolism and increases neuronal viability under stress conditions (Fruhbeis et al., 2013). It will be interesting to see in future studies if exosomes protect axons selectively from such insults.

The exosomal transfer of RNA species is consistent with a recent study demonstrating transport of 5-Bromouracil (BrU) labeled RNA from SCs into axons which colocalizes with actin and myosin after axon transection (Sotelo et al., 2013). Direct association between these components was shown using Fluorescence Resonance Energy Transfer (FRET) analysis (Canclini et al., 2013). In accord with the hypothesis that actin and myosin drive this RNA transfer, such transport appears mitigated in myosin5a knockout mice (Sotelo et al., 2013). There is also evidence for occasional vesicular transport of entire ribosomes from SCs into peripheral axons, and this transport appears strongly increased in injured axons (Court et al., 2008, 2011). This suggests the fascinating possibility that EG supply axons transcellularly with the entire set of machinery for production of axo-protective constituents locally. In fact, axons are autonomous regarding protein translation to some extent, as ribosomes, mRNA and obligatory translation regulators can be found in distal axons (Holt and Bullock, 2009; Twiss and Fainzilber, 2009). These components may be transported into the long axon all the way from the distant neuronal cell body, and also transferred from the nearby flanking glia for accelerated delivery. It is appealing to speculate that axonal translatome components described as axonal stability or survival factors such as Impa1, LaminB2 and Bcl-w could be transferred from EG (Andreassi et al., 2010; Yoon et al., 2012; Cosker et al., 2013).

These studies lend support for the concept that EG maintain axons by releasing exosomes and other vesicles. Our understanding of the mechanisms regulating the release, transport, and axonal uptake of these vesicles is still in its infancy. So far it is unknown if SC-derived exosomes or similar vesicles (Chen et al., 2012a; Zhu et al., 2013a) can be internalized by peripheral axons, and exert protective functions. Since these vessels are known to carry small molecules including RNA, macromolecular complexes and even entire organelles, they could impact axon integrity on various mechanistic levels. Future studies employing genetic mouse models and compartmentalized systems should determine the effects of disturbed vesicle secretion from EG on axonal integrity. Such work will also help to understand if exosomes are positive or negative regulators of myelination (Bakhti et al., 2011). If vesicular transfer proves axo-protective, the next step will be the identification of the components crucial for this function.

## Support of axons by ensheathing glia implicating intermediate energy metabolic pathways

The study of intermediate metabolism in the neurosciences has been often eclipsed by other subjects in the last decades. Metabolism recently started to experience a renaissance in light of new methodology, the importance for pathophysiology in man, and compelling evidence that energy metabolism in neurons and glia is closely linked. Additionally, the implication of crucial metabolic enzymes (i.e., Nmnat) and mitochondrial function in regulating axon integrity makes this field particularly appealing. There is no doubt that all anabolic functions are sustained by properly regulated energy metabolism, that is the conversion of carbohydrates and other fuels in order to generate ATP. Thus, it is obvious that the outlined support functions of EG can be impaired by defective energy metabolism. Furthermore, very specific metabolic functions in EG may support axons directly, as exemplified in the prevailing energy transfer model between astrocytes and neurons (astrocyte-neuron lactate shuttle) (Pellerin and Magistretti, 1994; Belanger et al., 2011). Given the intimate relationship between axons and EG (Figures 1, 2), a metabolic cooperativity seems plausible to maintain the EG-axon unit.

Recent work and novel genetic mouse models gave astounding initial insight into the consequence of metabolic defects in EG for axonal integrity both in the PNS and CNS. Disruption of mitochondrial respiration in SCs by conditional deletion of the mitochondrial transcription factor A gene *(Tfam)* or the *COX10* gene does not interfere with SC survival, but leads to demyelination and axonal degeneration (Viader et al., 2011; Funfschilling et al., 2012). In the CNS, partial inactivation of fatty-acid β-oxidation by inhibition of peroxisome biogenesis in OLGs is accompanied by white matter axon degeneration (Kassmann et al., 2007, 2011). As marked demyelination is observed in both models, and pronounced neuroinflammation in the latter, it remains possible that at least part of the axon loss is directly due to proinflammatory signals (Nave, 2010a). Thus, further hypometabolic EG mutants, free from such confounding effects, will be crucial to ascertain the importance of intermediate energy metabolism components for axon support.

The conserved sirtuin family of NAD^+^-dependent deacetylases (SIRT1-7) is known to modulate a variety of metabolic processes ranging from glycolysis to fatty acid oxidation in mitochondria (Yamamoto et al., 2007; Haigis and Sinclair, 2010). Based on its prominent reduction in the aforementioned PLP mutant mouse model with axonal degeneration, the cytosolic sirtuin member SIRT2 in OLGs may play an axon-supportive role (Werner et al., 2007). Moreover, arguing for a metabolic role of SIRT2 in SCs, this sirtuin was identified in gene expression profiling experiments as highly abundant protein in nascent SCs (Nagarajan et al., 2002). Surprisingly, SC-restricted SIRT2 knockout mice do not show axon degeneration even if aged, despite metabolic dysregulation in SCs (Beirowski et al., 2011). Possible explanations for this result in conjunction with the other studies may be that metabolic dysregulation in SIRT2-depleted SCs is not sufficiently severe to trigger axon loss, or that it is not confined to specific metabolic targets that are important for axonal survival. In light of these data it will be interesting to examine the consequences of SIRT2 ablation in OLGs for long-term structural axon integrity.

The idea that glia may provide specific energetic substrates to axons that are essential for axonal function and integrity has been expressed by various researchers in the last decades (Spencer et al., 1979; Hargittai and Lieberman, 1991; Ransom and Fern, 1997; Wender et al., 2000; Tachikawa et al., 2004), but only recently has it been revived and expanded (Nave, 2010a,b; Funfschilling et al., 2012; Lee et al., 2012; Morrison et al., 2013). Clues for relevant metabolites came from studies investigating their role for axonal electric function under stress conditions. Monocarboxylates (glucose and its intermediates) are the primary energy sources in the nervous system and are utilized to fuel neuronal activity primarily via oxidative metabolism (Belanger et al., 2011). Lactate has gained particular attention because it was proposed to be taken up and oxidatively metabolized by axons from surrounding glia that produce it from glucose (or glycogen) (Vega et al., 2003; Brown et al., 2004, 2005, 2012; Tekkok et al., 2005). This transport was proposed to be mediated through specific monocarboxylate carrier isoforms (MCTs), of which MCT2 is predominantly localized on axons, and MCT1 on ad-axonal glial cells (Tekkok et al., 2005; Rinholm et al., 2011). Indeed, recent *in vivo* data suggested that the reduced expression of the lactate transporter MCT1 in OLGs causes axon damage (Lee et al., 2012). In contrast, impairment of mitochondrial respiration in OLGs would not be expected to result in axon degeneration, since this should not directly interfere with glycolytic lactate production and transport via MCT transporters. Consistent with this line of reasoning, COX10 deficient OLGs produce normal or even increased levels of lactate, and thus were not associated with axonal degeneration (Funfschilling et al., 2012). It remains unclear why PNS axon degeneration is triggered by mitochondrial perturbation in SCs, but the mechanisms governing metabolic interactions of PNS axons and SCs may differ from that in CNS fibers (Brown et al., 2012; Evans et al., 2013). Also, as mentioned above, the accompanying demyelination phenotype and neuroinflammation are likely to contribute to axonal destabilization in this model. It will be interesting to study in future if EG release other axon-protective metabolites as has been suggested in a different context (Vega et al., 1998; Tachikawa et al., 2004; Belanger et al., 2011). By the same token, it is not entirely clear if lactate is the only metabolite transported via axonal and EG-localized MCTs, as pyruvate and ketone bodies can also be shuttled along this route (Morrison et al., 2013).

Leaving these aspects about myelination and lactate aside, it has been proposed that the myelin sheath itself could contribute to metabolic support for large axons by ectopic combustion of glucose and ATP generation mediated by mitochondria-independent oxidative phosphorylation (Ravera et al., 2009, 2013a; Adriano et al., 2011; Morelli et al., 2011). In support of this hypothesis, glycolytic and tricarboxylic acid cycle (TCA cycle) enzymes including ATP synthase have been recently identified in isolated myelin vesicles (IMV) from forebrain preparations (Ravera et al., 2009, 2011, 2013a). These vesicles display a transmembrane electrochemical proton gradient and are capable of consuming oxygen when stimulated with pyruvate. There seems to also be evidence for aerobic ATP synthesis in IMV, which can be blocked by various mitochondrial redox complex inhibitors (Ravera et al., 2009; Rinholm et al., 2011). Notably, oxygen consumption was impaired in IMVs from adult CMT model rats that display axonal loss in the PNS (Ravera et al., 2013b), supporting the possibility that myelin respiratory chain disruption contributes to axon loss in disease. However, the notion that myelin could serve as an ATP generator has been recently challenged by several theoretical considerations based on what is known about electrophysiological parameters of EG and the configuration of the ATP synthase complex (Harris and Attwell, 2013). According to these authors, it is most likely that the presence of respiratory chain components in myelin preparations reflect contaminations by mitochondrial membranes.

In contrast to myelin’s typically salutary characterization, myelination also has been proposed to be unfavorable to axons because it may hinder metabolic exchange due to the peculiar cytoarchitecture of compact myelin sheaths (Edgar et al., 2009; Nave, 2010b). Consistent with this, axonal degeneration is observed in EG-specific *Phosphatase and tensin homolog (PTEN)* knockout mice with hyperactivation of PI3 kinase leading to substantially increased myelin sheath thickness (Goebbels et al., 2010, 2012; Harrington et al., 2010). An alternative explanation may be that physical compression of axons by exuberant glial growth and excess myelin results in injury. In addition, it is also conceivable that in these mice metabolic or signaling alterations lead to axonal compromise because of aberrant PI3 kinase signaling as a direct consequence of *PTEN* deficiency in EG.

In sum, recent studies provide growing evidence that intermediate metabolic pathways in EG have a crucial role in the non-cell-autonomous support of structural axonal integrity. Future work, making use of novel mouse models with metabolic imbalances that mimic altered metabolic states in aging, diet-associated disease, and neurodegeneration, while avoiding confounding neuroinflammatory signals, will be important for determining the relevance of the identified pathways. Additionally, emerging literature from the last year suggests that EG are metabolically coupled to long axons by conveying small metabolites such as lactate into the axoplasm to fuel axonal energetic needs. As highly sensitive optical lactate biosensors together with other tools are becoming available for real-time and single-cell-resolution imaging (San Martin et al., 2013), it should be possible to visualize lactate trafficking between EG and axons in future. Finally, *in vivo* investigations with conditional ablation of carriers for lactate and other carbohydrates in axons and EG are warranted to test above model.

## Conclusions and future perspective: linking glial support of axons with pathways regulating axonal self-destruction in disease

**Figure 3:**
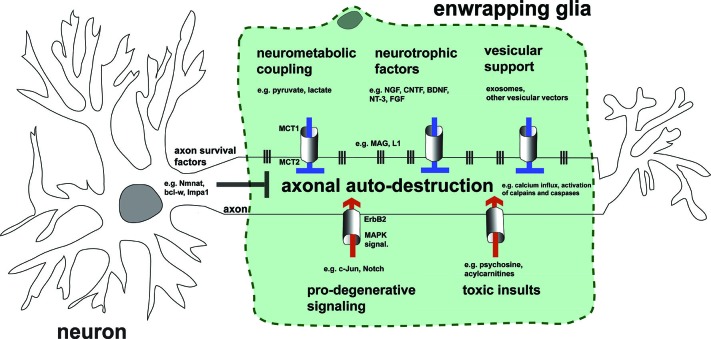
**Hypothetical model summarizing impact of EG on axonal integrity by the mechanistic themes discussed.** Preservation of healthy axons is controlled by the cooperative action of both the neuron and adjacent glia by blocking endogenous axonal auto-destruction. Examples of neuronal mechanisms blocking this program(s) include somatic delivery of the putative axonal survival molecule Nmnat2 into axons, or the local translation of axonal maintenance factors such as Bcl-w. On the other hand, glia inhibit axonal death by transfer of metabolic substrates, neurotrophic factors, and vesicular shuttles (upper glia portion). This transfer is mediated by specific transport mechanisms represented by columns between glia and axon. Vertical lines embody adhesion mechanisms that ensure correct apposition and formation of nutritive channels between glia and axon. Under pathological conditions glia may also contribute to axonal auto-destruction by activating pro-degenerative signaling, releasing toxic substances, and loosening contact to axons (lower glia portion). Note that the myelin membrane of EG is uncoiled in the illustration and myelination thus not represented.

The neuroscientific community has long been interested in the intimate relationship between axons and their EG, but the role of these glia for axon integrity has been neglected in favor of neuron-autonomous mechanisms of axon degeneration. As evident from the data reviewed here, EG can be considered active players with instructive functions during degeneration, instead of passive bystanders. At the same time, EG provide support for structural axon integrity by means of their tight association to axons, release of trophic substances, and metabolic coupling with axons. In other words, it becomes clear that long axons in real circumstances cannot exist without their accompanying glia throughout life. Powerful evidence indicates that dismantling of damaged axons is controlled by an auto-destructive cascade akin to apoptosis (but independent of somatic cell death mechanisms), which seems to be continuously kept inactive in healthy axons. Hence, it seems legitimate to speculate that prevention of this axonal death program is a cooperative mechanism between neurons and EG (Figure 3, upper glia portion). The data presented lay a foundation for a conceptual framework addressing the question of how the described EG functions could contribute to block self-destruction of axons. While it seems more straightforward in this perspective to connect the release of neurotrophic factors from EG to this mode of axonal protection, establishing the same links to other released substances and small metabolites is certainly a more formidable task. Such compounds released by EG and internalized into axons could directly or indirectly block putative signaling cascades promoting axonal degeneration (e.g., by modulating the trafficking and concentration of the axonal survival factor Nmnat2). Alternatively, EG-derived substances could impinge on other checkpoints influencing axonal integrity, such as mitochondrial energetics, axonal sodium and calcium levels, or regulation of axonal transport mechanisms. Of course, these alterations may also feed back and influence the levels and distribution of primary axonal survival factors. This scenario perhaps appears more relevant for the EG-to-axon transfer of small energy substrates that are likely to affect many of these functions by fuelling mitochondrial ATP generation in axons. Indeed, impairment of mitochondrial bioenergetics (Ferri et al., 2006), abnormal calcium homeostasis (Parone et al., 2013) as well as perturbations in axonal transport (Marinkovic et al., 2012) are hallmarks of ALS models in which axon degeneration is a primary pathological feature (Fischer et al., 2004). Intriguingly, early molecular abnormalities in OLGs, consistent with perturbed metabolic coupling including reduced levels of MCT1 on oligodendroglia have been recently observed in both mouse ALS models and in human ALS tissues (Lee et al., 2012; Kang et al., 2013; Philips et al., 2013). This supports an increasing perception that progression of motor axon degeneration in ALS is determined by non-cell-autonomous effects. The reduced content of MCT1 on oligodendroglia and their cellular precursors may be a direct consequence of glial mutant SOD1 expression (Philips et al., 2013). An exciting link between perturbed neurometabolic coupling and axon degeneration may be even more apparent in the large group of metabolic peripheral neuropathies with polygenic etiologies, and most prominently in those associated with diabetes. Axon degeneration is a cardinal feature in diabetic peripheral neuropathy (DPN) and it remains obscure why predominantly sensory fibers, both unmyelinated and myelinated, are affected early in diabetic axonopathy and in its animal models (Ramji et al., 2007; Said, 2007; Zochodne, 2007). In principle similarly as above, perturbed neuronal mitochondrial bioenergetics, defective calcium homeostasis, and axonal transport deficits in peripheral nerves are documented in DPN models, although the underlying mechanisms are only poorly understood (Fernyhough and Calcutt, 2010; Chowdhury et al., 2013). Remarkably, in addition to characteristics such as decreased release of neurotrophic factors, accumulation of detrimental compounds (e.g., polyols, reactive oxygen species), microvascular complications, and inflammatory changes, a series of metabolic alterations in SCs have been proposed in the pathogenesis of DPN, and it is possible that these directly contribute to the detrimental effects on axon integrity (Eckersley, 2002; Kennedy and Zochodne, 2005; Sango et al., 2011; Chowdhury et al., 2013; Hinder et al., 2013; Kim et al., 2013). This and further examples support the idea that pathogenically relevant metabolic perturbation in EG might be a commonality in a number of chronic axonopathies associated with abnormal cell and whole-body metabolism. The differential metabolic and bioenergetic requirements for the maintenance of large and small, as well as myelinated vs. unmyelinated fibers, could provide a mechanistic basis for the well-known but poorly understood susceptibility of distinct classes of axons in these conditions (i.e., large motor axons in ALS versus small sensory axons in DPN).

On the contrary, in neurodegenerative situations EG may also directly perpetrate axonal insults by activation of pro-degenerative signaling or by release of axo-toxic compounds (or both). This may equally induce active axon degeneration programs (Figure 3, lower glia portion). In light of evidence that accumulation and release of abnormal metabolic intermediates from SCs and OLGs can compromise axons (e.g., psychosine release) (Cantuti Castelvetri et al., 2013; Viader et al., 2013), it is conceivable, although speculative at this point, that EG may also provide axonal support by continuous ad-axonal detoxification pathways. This idea is consistent with observations of detoxification mechanisms by superoxide dismutase activity in SCs, again in the context of mutant SOD1-mediated ALS (Wang et al., 2012b).

In conclusion, there is now a mounting consensus that axon degeneration is not simply due to passive decay of axonal components as a consequence of disconnection from the cell body. Specific axonal molecules are required for axonal survival, and these could be direct inhibitors of one or more possible auto-destructive pathways in axons that are mechanistically independent of somatic death. A great challenge in the future will be to integrate the question of how the model of trophic axonal support by EG leads to continuous inhibition of these auto-destructive axon degeneration programs. Moreover, and not mutually exclusive, mechanistic studies are needed to precisely determine how toxic signals from glia (instead of decreased salutary signals) can activate axonal degeneration programs. In addition to novel conditional mutants in the whole-animal setting, valuable tools that could cast light on these questions include compartmentalized microfluidic systems in which specific interactions between axons from distinct neuronal types and EG can be investigated, together with methods to globally characterize released molecules (Hosmane et al., 2010; Park et al., 2012; Shi et al., 2013). To attain a comprehensive and rounded view of how nerve and white matter integrity is maintained, and how aberrations in glia bring about axon degeneration, it is essential not to lose sight of continuous axon-glia communication. This may open the way to multimodal therapeutic approaches to stabilize injured axons in disease.

## Conflict of interest statement

The author declares that the research was conducted in the absence of any commercial or financial relationships that could be construed as a potential conflict of interest.
